# Supplementary material for: The Appearance of Osteomyelitis of the Foot and Disseminated Subcutaneous Abscesses During Treatment for Disseminated Tuberculosis Infection in an Immunocompetent Patient: Case Presentation of a Paradoxical Reaction and Literature Review
Source: Infect Dis Rep. 2025 May 2;17(3):46. doi: 10.3390/idr17030046 (PMC12101366; doi:10.3390/idr17030046)
Supplement: Supplementary file 1 [file idr-17-00046-s001.zip › idr-3516173-supplementary.pdf]

AUTHORS	GENDER	AGE	CLINICAL STATUS	CLINICAL PRESENTATION	INSTRUMENTAL EXAMS	TUBERCULOSIS CLASSIFICATION	MICROBIOLOGICAL DIAGNOSIS	THERAPY	ONSET	PARADOXICAL REACTION	DIAGNOSTIC TEST	PARADOXICAL REACTION CLASSIFICATION	THERAPY	OUTCOME
Alharbi et al. 2024 [1]	F	19	Malnutrition (BMI: 19)	Cervical lymphadenopathy fistulized, cough, fever and weight loss	Chest-XR: left upper lobe opacities<br><br>Neck–chest CT: left supraclavicular necrotic lymph nodes and cavitating consolidation in the left upper lobe with few scattered satellite nodular opacities	Pulmonary tuberculosis<br><br>Lymph node tuberculosis	Material lymph node: smear, negative; PCR (GeneXpert MTB/RIF method), positive for Mycobacterium tuberculosis Culture positive for Mycobacterium tuberculosis	300 mg isoniazid, 600 mg rifampin, 800 mg ethambutol and 1000 mg pyrazinamide for 10 days<br><br>Suspension for 9 days due to liver enzyme increase<br><br>Ethambutol, moxifloxacin, rifampin and isoniazid	4 weeks	Skin abscess New lymphadenopathy (submandibular) Worsening of pre-existing lymphadenopathy	Left-hand XR: intact bone anatomy with absence of osteomyelitis<br><br>Aspiration pus: routine bacterial culture negative AFB culture negative AFB smear negative PCR (Genexpert MTB/RIF method) positive for Mycobacterium tuberculosis	Cutaneous tuberculosis<br><br>Lymph node tuberculosis (LNTB)	Isoniazid, rifampin, moxifloxacin and ethambutol for 2 months,<br><br>then<br><br>isoniazid, rifampin and moxifloxacin for 10 months	Follow-up six months post therapy completion showed improvement, without disease recurrence
Samad et al. 2023 [2]	M	42		Shortness of breath, asthenia and weight loss	Chest XR: diffuse bilateral opacity<br><br>Angio-TC: diffuse micronodules of hematogenous spread	Miliary tuberculosis	Bronchoscopy: PCR positive<br><br>Lumbar puncture: meningitis, PCR negative, culture negative Trans jugular hepatic biopsy: non caseating granuloma histopathology	Spiramycin, cefotaxime, linezolid and amikacin (for respiratory failure followed by shock), followed by de-escalation to cefaxoline for identification of MRSA sovrainfection, then ceftazidime due VAP due to pseudomonas<br><br>After tuberculosis diagnosis: Rifampicin 600 (10 mg/Kg) iv; Isoniazid 200 mg (3.5 mg /kg) iv; Ethambutol 1200mg (20 mg/kg) iv Pyrazinamide (30 mg/kg) os Prednisolone 1 mg/kg (for significant inflammation)	29 days	Left hemiparesis and hyperactive deep tendon reflexes	MRI: multiple lesions compatible with tuberculoma, left parietal hematoma surrounded by edema, moderate hydrocephalus with sign of resorption, arachnoiditis and lumbar epiduritis Cerebrospinal fluid (CSF): negative for tuberculosis,<br><br>Fundoscopic exam: no papilledema	Meningeal tuberculosis	Increased rifampicin to 30 mg/Kg (reduced to 20 mg/kg after 27 days, then oral): 12 months, isoniazid 5 mg/kg (12 months), ethambutol switched to levofloxacina 500 mg reintroduced for 6 months dexamethasone 25 mg (tapering after 1 month) infliximab 300 mg every 15 days	Resolution of miliary opacity; disappearance of observed pontine lesions, residual sequelae of the parietal hematoma and resolution of its perilesional edema
Volpe-Chaves et al. 2020 [3]	M	39	History of alcoholism	Cough, fever, weight loss, mental confusion and stiff neck	Chest XR: alveolar–interstitial consolidations in the upper lobes	Pulmonary tuberculosis<br><br>Meningeal tuberculosis	Sputum research and culture positive for Mycobacterium tuberculosis<br><br>Cerebrospinal fluid: PCR (Gene Xpert MTB/RIF) positive for Mycobacterium tuberculosis	Isoniazid 75 mg, Rifampin 150 mg, Ethambutol 275 mg Pyrazinamide 400 mg and 4-mg Dexamethasone IV (0.3mg/kg/d) until hospital discharge, then Prednisone os with a total treatment time of <8 weeks	2 months	Fever and abdominal and lumbar pain	Lumbosacral spine MR: signal alteration and a heterogeneous enhancement by contrast medium from T8 to T11, reduction of the vertebral bodies height of T10 and T11 and collapse of the respective intervertebral space<br><br>Abscess drainage material: direct BAAR negative, PCR (Gene Xpert MTB/RIF) positive for Mycobacterium tuberculosis Culture positive for Mycobacterium tuberculosis	Osteoarticular tuberculosis	CT-guided abscess drainage<br><br>75 mg isoniazid, 150 mg rifampin, 275 mg ethambutol and 400 mg pyrazinamide for 12 months	Complete clinical improvement and follow-up with programmed neurosurgery for repair of the bone fracture

Shinga et al. 2019 [4]	M	26		Chronic cough, evening fever, deterioration of general condition and weight loss	Chest XR: right pleuro-pneumopathy	Pleuro-pulmonary tuberculosis	Pleural fluid: GeneXpert negative and culture negative<br><br>Chemical–physical: protein, 40 g/l; elements, 320 mm/mm3; lymphocytic 100%	Rifampin, Ethambutol, Isoniazid, Pyrazinamide for 2 months<br>Rifampin and Isoniazid for 4 months	1 month	Abdominal pain, vomiting, cessation of feces and gas	Abdominal XR: hydro-eric images<br><br>Abdominal-CT: small intestine occlusive syndrome<br><br>Anatomopathological examination of surgical specimen: area of eosinophilic and acellular necrosis surrounded by epithelioid cells and a lymphocytic crown associated with Langerhans giant cells without signs of malignancy (= caseofollicular intestinal tuberculosis)	Intestinal tuberculosis	Surgical removal of intraperitoneal mass<br><br>Rifampin, ethambutol, isoniazid and pyrazinamide continued for 6 months	Resolution of all symptoms
Kabra et al. 2019 [5]	M	34		Fever, sore throat, cough and generalized weakness	Chest XR: prominent left hilum<br><br>Chest CT: multiple discrete and confluent necrotic lymph nodes in the right paratracheal, bilateral tracheobronchial, subcarinal, left para-aortic and left hilar regions and multiple opacities in the apical segment of the lower lobe of the left lung	Pulmonary tuberculosis<br><br>Lymph node tuberculosis	Sputum AFB positive GeneXpert for Mycobacterium tuberculosis positive, culture positive	Rifampin, Ethambutol, Isoniazid, Pyrazinamide	2 months	Worsening cough, sore throat and anorexia	Chest CT: increase in the number of nodal stations and nodular lung lesions with hepatosplenic involvement<br><br>Aspiration (FNAC) right paratracheal and left hilar lymph nodes:<br>AFB smear positive, GeneXpert non detected, culture negative	Worsening lymphadenitis tuberculosis<br><br>Hepatic tuberculosis<br><br>Splenic tuberculosis	Oral prednisolone (tapered over 8 weeks)	Chest CT: decrease in number and size of mediastinal lymphadenopathy with interval resolution of lung parenchymal nodules. Completed first-line ATT and recovered without any further complications.
Kabra et al. 2019 [5]	M	63	Diabetes mellitus, systemic arterial hypertension and psoriasis	Fever, cough, chest pain and dyspnea	Chest XR: increased cardiothoracic ratio<br>Echo: moderate concentric left ventricular hypertrophy<br><br>Chest/abdomen: loculated fluid density in the anterior mediastinum with enlarged partially necrotic lymph nodes and increased vascularity and bilateral pleural effusion (left>right)	Lymphadenitis tuberculosis	FNAC: AFB stain positive, AFB culture positive	Rifampin, Ethambutol, Isoniazid, Pyrazinamide	2 months	Chest heaviness, shortness of breath, cough and fever	Chest CT: increase in size and extent of the mediastinal loculated collection<br>Chest–abdomen CT: revealed bilateral moderate pleural effusion (left > right), moderate to significant pericardial effusion and heterogeneous-enhancing inflammatory changes in anterior mediastinum perivascular space in retrosternal location<br><br>2D echo: early effusive constrictive pericarditis with mild pericardial effusion	Tuberculous pleural effusion<br><br>Tuberculous pericarditis	Moxifloxacin and amikacin<br>Oral corticosteroid	Paradoxical upgradation response in non-HIV tuberculosis: report of two cases

Neki et al. 2017 [6]	NR	NR		Fever with chills, night sweats and cough	Chest-XR: normal<br><br>Chest-CT: enlargement of hilar lymph nodes with necrotic areas	Lymph node tuberculosis	NR	Antitubercular treatment	25-27 days	Severe cough and severe dyspnea	Chest XR: massive hilar lymphadenopathy<br><br>Chest CT: enlarged mediastinal lymph nodes, involvement of newer lymph nodes and collapse of the lower part of the right lung<br><br>EBUS: PCR positive for Mycobacterium	Pulmonary tuberculosis<br><br>Lymphadenitis tuberculosis	Short tapering course of oral corticosteroids<br><br>Continued ATT therapy	Clinical improvement, CBNAAT negative and chest CT showed resolution of lymphadenopathy and collapse
Chaskar et al. 2015 [7]	M	35		Fever, weight loss, loss of appetite and painless lump on right side of chest	Chest-XR: normal<br><br>Ultrasonogram: hypoechoic lesion with dense internal echoes and minimal fresh fluid with dense echoes	Musculoskeletal tuberculosis	NR	Antitubercular treatment	3 weeks	Swelling gradually increased	Aspiration abscess fluid: Ziehl Neelsen (ZN) stain positive for acid fast bacilli morphologically resembling Mycobacterium tuberculosis and culture positive for Mycobacterium tuberculosis	Increase in lump size	Continued ATT therapy	Follow-up after two months of ATT showed clinical improvement

Tab 1. *Cases of paradoxical reactions during anti-tuberculosis therapy in immunocompetent adults suffering from tuberculosis described in the literature from 2014 to 2024.*

References

1. Alharbi, A.; Aljahdali, A.; Ahamed, M.F.; Almarhabi, H. Left hand abscess as a paradoxical reaction during treatment of disseminated tuberculosis in immunocompetent patient: Case report and review of literature. *BMC Infect. Dis.* **2024**, *24*, 1186. https://doi.org/10.1186/s12879-024-10077-w. PMID: 39434028; PMCID: PMC11492750.
2. Samad, M.; Dallevet, C.A.; Tandjaoui-Lambiotte, Y.; Bourgarit, A.; Jaquet, P. Spectacular Improvement of Paradoxical Reaction in Tuberculosis after Tumor Necrosis Factor-Alpha Antagonist Therapy. *Cureus* **2023**, *15*, e50596. https://doi.org/10.7759/cureus.50596. PMID: 38222121; PMCID: PMC10788139.
3. Volpe-Chaves, C.E.; Lacerda, M.L.G.G.; Castilho, S.B.; Fonseca, S.S.O.; Saad, B.A.A.; Franciscato, C.; Tibana, T.K.; Nunes, T.F.; Venturini, J.; Oliveira, S.M.D.V.L.; et al. Vertebral tuberculosis as a paradoxical reaction to the treatment of pulmonary and meningeal tuberculosis in an immunocompetent patient: A case report. *Medicine* **2020**, *99*, e20012. https://doi.org/10.1097/MD.0000000000020012. PMID: 32481268; PMCID: PMC7249907.
4. Shinga, B.W.; Dièye, A.; Badiane, N.M.D.; Lakhe, N.A.; Diallo, V.M.C.; Mbaye, K.D.; Ka, D.; Badiane, A.S.; Diouf, A.; Déguénonvo, L.F.; et al. Tuberculose intestinale révélée par une occlusion intestinale aigüe au cours d'une réaction paradoxale au traitement anti-tuberculeux chez un patient immunocompétent: À propos d'un cas et revue de la littérature [Intestinal tuberculosis revealed by acute bowel obstruction during paradoxical reaction to antituberculosis treatment in an immunocompetent patient: About a case and literature review]. *Pan Afr. Med. J.* **2019**, *32*, 173. (In French). https://doi.org/10.11604/pamj.2019.32.173.17893. PMID: 31303942; PMCID: PMC6607298.
5. Kabra, M.N.; Kunapareddy, T. Paradoxical upgradation response in non-HIV tuberculosis: Report of two cases. *Indian J. Med. Microbiol.* **2019**, *37*, 450–453. https://doi.org/10.4103/ijmm.IJMM_18_242. PMID: 32003352.
6. Neki, N.S.; Joshi, N.; Shergill, G.S.; Singh, A.; Meena, N.K.; Kaur, A.; Dhanju, A.S.; Vaid, A.; Kausahal, D. Paradoxical Reaction in an Immunocompetent Internal Medicine Resident Suffering from Tubercular Lymphadenitis. *Int. J. Health Sci. Res.* **2017**, *7*, 388–392.
7. Chaskar, P.; Rana, G.; Anuradha Duggal, N.; Arora, J. Treatment paradox in musculo-skeletal tuberculosis in an immunocompetent adult male; a case report from a tertiary care hospital. *J. Clin. Diagn. Res.* **2015**, *9*, DD01–DD02. https://doi.org/10.7860/JCDR/2015/10943.5749. PMID: 26046019; PMCID: PMC4437066.
